# Modeling transmission of avian influenza viruses at the human-animal-environment interface in Cuba

**DOI:** 10.3389/fvets.2024.1415559

**Published:** 2024-07-11

**Authors:** Damarys de las Nieves Montano Valle, John Berezowski, Beatriz Delgado-Hernández, Adrian Quintana Hernández, María Irian Percedo-Abreu, Pastor Alfonso, Luis Pedro Carmo

**Affiliations:** ^1^Epidemiology Group, National Center for Animal and Plant Health (CENSA), World Organisation for Animal Health (WOAH) Collaborating Center for the Reduction of the Risk of Disaster in Animal Health, San José de las Lajas, Cuba; ^2^Center for Epidemiology and Planetary Health, Scotland's Rural College, Inverness, United Kingdom; ^3^Veterinary Public Health Institute, Vetsuisse Faculty, University of Bern, Liebefeld, Switzerland; ^4^National Center of the Protected Areas, Playa, Havana, Cuba; ^5^Norwegian Veterinary Institute, Ås, Norway

**Keywords:** One Health, integrated surveillance system, disease introduction, disease transmission, risk factors, wild birds, livestock

## Abstract

**Introduction:**

The increasing geographical spread of highly pathogenic avian influenza viruses (HPAIVs) is of global concern due to the underlying zoonotic and pandemic potential of the virus and its economic impact. An integrated One Health model was developed to estimate the likelihood of Avian Influenza (AI) introduction and transmission in Cuba, which will help inform and strengthen risk-based surveillance activities.

**Materials and methods:**

The spatial resolution used for the model was the smallest administrative district (“Consejo Popular”). The model was parameterised for transmission from wild birds to poultry and pigs (commercial and backyard) and then to humans. The model includes parameters such as risk factors for the introduction and transmission of AI into Cuba, animal and human population densities; contact intensity and a transmission parameter (β).

**Results:**

Areas with a higher risk of AI transmission were identified for each species and type of production system. Some variability was observed in the distribution of areas estimated to have a higher probability of AI introduction and transmission. In particular, the south-western and eastern regions of Cuba were highlighted as areas with the highest risk of transmission.

**Discussion:**

These results are potentially useful for refining existing criteria for the selection of farms for active surveillance, which could improve the ability to detect positive cases. The model results could contribute to the design of an integrated One Health risk-based surveillance system for AI in Cuba. In addition, the model identified geographical regions of particular importance where resources could be targeted to strengthen biosecurity and early warning surveillance.

## 1 Introduction

From October 2021 until the time of writing, an unprecedented number of highly pathogenic avian influenza outbreaks have been reported in different regions of the world. The disease threatens global food security and the livelihoods of households dependent on poultry (1, 2). At present, an increasing number of cases of H5N1 Avian Influenza (AI) have been reported even in several species of mammals, both terrestrial and aquatic, causing increased morbidity and mortality and raising concerns about the threat to domestic animal and wildlife health, biodiversity and potentially to public health (3). Exponential growth of the human population and its geographic expansion have increased the importance of the human-animal-environment interface. Surveillance of domestic poultry and farm workers has shown that avian influenza viruses (AIVs) are constantly evolving and are able to cross species barriers and infect mammals (1, 4–6), which raises concerns about the potential for AIVs to spark a human pandemic. Therefore, continuous improvement of surveillance and control of AI has become a global priority, and early detection of outbreaks with pandemic potential has become an important public health priority.

Despite the persistence of siloed thinking in disease surveillance and control activities, collaborative One Health initiatives have emerged. These have been mainly focused on the prevention of zoonotic diseases and antimicrobial resistance (7). One Health Surveillance Systems (OHSS) are essential for promoting a holistic, integrated approach to disease prevention and mitigation. OHSS requires that collaborative efforts between health sectors (human, animal, plant, food safety, wildlife and environment) are established during the surveillance process (8). Integrating data from multiple sources and knowledge from various disciplines in an OHSS can help to identify emerging health threats earlier, track the spread of disease, and inform evidence-based policies and interventions to promote health and wellbeing across sectors.

Surveillance of poultry and livestock plays a critically important role in identifying changes in the risk of transmission of influenza viruses to humans. Disease surveillance enables the early detection of infections by AI and provides data on the evolution of AIVs, which is essential for identifying and assessing changes in virus virulence and transmissibility to humans. Surveillance data are used to understand the dynamics of virus transmission, which contributes to the development and implementation of preventive measures such as biosecurity protocols, vaccination programmes and control strategies to reduce the risk of transmission to humans, especially in areas where close human-animal contact is frequent (9).

Simulation models that predict the spread of disease outbreaks play an important role in epidemic control. Understanding where a disease is most likely to spread constitutes valuable information for developing risk-based surveillance and targeted disease prevention and control strategies (10). Several models have been developed to predict the risk and spread of AIV within poultry populations (11–15). The outputs from these models can play an important role in informing surveillance activities by identifying high-risk regions and populations for targeted surveillance (16).

Migratory waterfowl constitute the greatest risk for the introduction of AIV into Cuba. Infection with Highly Pathogenic Avian Influenza Viruses (HPAIVs) are an exotic disease of poultry in Cuba. However, HPAIVs are major threat to the Cuban poultry production sector, which plays an important role in the domestic production of protein of animal origin and, consequently, in food security. The aim of this study was to develop a model using a One Health approach to estimate the relative risk of AIVs transmission at the human-animal-environment interface, that can be used to inform risk based surveillance for an avian influenza incursion in livestock and humans.

## 2 Materials and methods

### 2.1 Model summary

**Figure 1:**
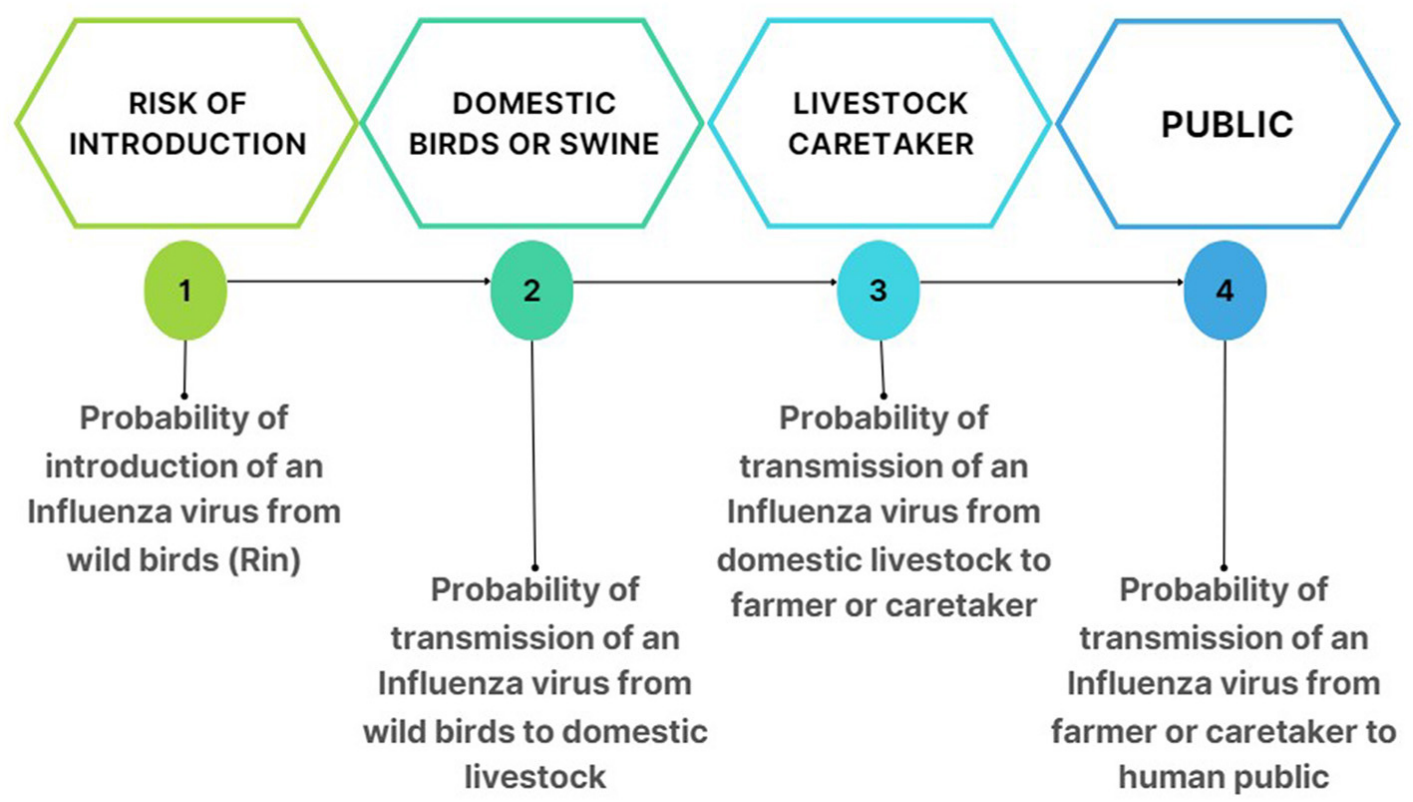
Pathways of influenza virus transmission for the domestic birds and domestic swine risk estimation models.

The aim of the overall model was to estimate the relative risk of AIV spillover at the human-animal-environment interface. Two separate models were developed, one for poultry and one for swine. The models consider migratory waterfowl, the main reservoir of AIVs (17, 18), to be the most likely source for the introduction of AIVs into Cuba. The transmission pathways included were: (1) from wild birds to domestic birds and from domestic birds to farmers/agricultural workers and then to the public, and (2) from wild birds to domestic swine and from domestic swine to farmers/agricultural workers and then to the public (Figure 1).

Transmission risk was assessed by incorporating specific information about risk factors associated with virus introduction and other parameters related to disease transmission among livestock and farm workers. An epidemiological risk equation was used as a function of the spatial density of each parameter, taking into account some concepts developed by Hill et al. (18) with some modifications.

It was assumed that for zoonotic transmission to occur there must be infected animals within the effective transmission range of susceptible humans, and that the number of contacts is proportional to the product of the number of infected animals and the number of susceptible humans. There must also be some transmission efficiency, which may vary depending on the extent and intensity of contact between animals and humans (e.g., commercial versus backyard poultry production). Intensity was assessed as an indicator of the level of opportunity for human exposure to influenza viruses. Other parameters used by Hill et al. (18), such as virus specific transmission components for specific influenza strains and the prevalence of specific strains in poultry, were not considered due to lack of data. The spatial resolution used for the risk assessment was the smallest administrative structure named “Consejo Popular.” The model was parameterized for poultry and swine to humans. Parameter estimates are presented in Table 1.

**Table 1:** Summary of parameter estimates.

**Parameter**	**Description**	**Mean (min, max)**
Rin_cp_	Introduction risk of influenza virus by wild birds for each “Consejo Popular” (cp)	1.3 × 10^−3^ (0, 9.3 × 10^−2^)
L_sp, j, cp_	**Animal densities by (1) species (sp): ducks, poultry, swine, (2) production type (j), (3) Consejo Popular (cp)**	
	Commercial ducks Commercial chickens Backyard chickens Commercial swine Backyard swine	6.6 × 10^−4^ (0, 0.13) 6.6 × 10^−4^ (0, 0.04) 6.6 × 10^−4^ (0, 0.05) 6.6 × 10^−4^ (0, 0.28) 6.6 × 10^−4^ (0, 0.16)
Cr_sp, j_	**Contact ratio between (1) production types:** commercial and backyard chickens, commercial ducks, commercial and backyard swine and (2) **to farm worker/farm owner. The following contact ratios were included in the model:**	
	Commercial chickens → farm worker Backyard chickens → farm owner Commercial ducks → farm worker Commercial swine → farm worker Backyard swine → farm owner	2.0 × 10^−4^ (7.9 × 10^−5^, 3.3 × 10^−4^) 0.05 (0.02, 0.09) 5.7 × 10^−4^ (1.6 × 10^−4^, 9.9 × 10^−4^) 0.10 (8.3 × 10^−3^, 0.13) 0.51 (0.04, 0.9)
**β**_sp, j_	**Transmission parameters for production type to farm worker/farm owner:**	
	Commercial chickens → farm worker Backyard chickens → owner Commercial ducks → farm worker Commercial swine → farm worker Backyard swine → owner/ farm worker	2.0 × 10^−7^ (1.16 × 10^−9^, 1.3 × 10^−6^) 1.6 × 10^−6^ (6.05 × 10^−8^, 5.6 × 10^−6^) 9.16 × 10^−7^ 1.54 × 10^−7^(5.4 × 10^−9^, 4.1 × 10^−7^) 4.76 × 10^−3^(5.3 × 10^−4^, 1.6 × 10^−2^)
Hp_cp_	Density of human population	1655.00 (3.07, 271173.23)

cp, Consejo popular; sp, Species; j, Production type.

### 2.2 Data processing and analysis

The R programming software (R software version 4.3.0) was used for data processing and model development. The geographic information system (GIS) QGis version 3.28.3 in the NAD 27 (3795) projection was used to manage the spatial layers. In order to unify the format of each layer, map data were converted into vectors, with the “Consejo Popular” as the unit of spatial resolution. The parameters and results obtained in each equation of the models were normalized between zero and one using the following formula:


$$\begin{array}{l} Z\;=\frac{X}{Sum\left(X\right)} \end{array}$$

Where Z is the normalized value of the variable X. The empirical cumulative distribution function (ECDF) was used to plot the data points in the sample from smallest to largest against their percentiles. The threshold for risk categorization (high or low) was chosen visually from the ECDF plot by identifying the numerical value that coincided with the point where the ECDF curve started to turn from a vertical to a horizontal direction.

#### 2.2.1 Sensitivity analysis

Sensitivity analysis (SA) was used for quantifying uncertainty in the model. Sampling-based methods using Monte Carlo simulations were used for SA. First, the scatter plots of the data were analyzed and non-linearity and non-monotonicity of the data were determined graphically. Uncertainty analysis was then carried out using the Sobol method. This is based on the decomposition of the variance of the model output and aims to determine the extent to which the variability of the model output depends on each of the input parameters, either a single parameter or an interaction between different parameters (19).

#### 2.2.2 Probability of introduction of an AIV by wild birds (Rin)

The most important risk for AIV introduction into Cuba is assumed to be through migratory waterfowl (20, 21). Previous multi-criteria analysis based on weighted criteria were carried out to identify and evaluate the different risk factors that could be associated with the introduction of avian influenza viruses into Cuba (11). The risk factors reported to be important (11, 21) were the number of permanent wetlands (Pw), rice fields (Rf) and important bird areas (IBAs), i.e. settlements areas where water birds feed in natural and artificial bodies of freshwater (22, 23) in a community. These same three risk factors were used in the models developed in our study. The National Center provided data for IBAs and Pw for each “Consejo Popular” for the Protected Areas (CNAP) in Cuba.

Each risk factor was represented on an individual map consisting of one layer in vector format. Each area was assigned a density value for the risk factor present, based on the density of the factor in the “Consejo Popular”. The thematic layers produced were unified by overlay and visualized on a single map using the equation for estimating the risk of introduction of AIVs (Rin) at the “Consejo Popular” level. In the equation, a weighted sum was chosen to define which factors were more highly associated with the introduction of the AIV.

In the weighted sum method, the value of each cell of the maps was multiplied by a weighting factor according to the relative importance of each risk factor. This semi-quantitative weighting is an important step in this method and it is based on a detailed analysis of each element from a literature review (11) and by consultation with experts in the field (21). The transformations, based on the area of each “Consejo Popular” and the weighting are shown below (Table 2).

**Table 2:** Weighting of the factors used to estimate the risk of avian influenza virus introduction into a Consejo Popular.

**Densities of risk factor**	**Acronym**	**Weight (w)**
Rice field (occupied area/km^2^)	D_Rf_	0.0919
Important wild birds areas (IBAs) (occupied area/km^2^)	D_IBAs_	0.0919
Permanent wetlands (occupied area/km^2^)	D_Pw_	0.0748

The final equation for estimating the Risk of AIV introduction (Rin) into one “Consejo Popular” is:


(1) $$\begin{array}{l} Rin_{cp}=\left(D_{Rf}\times \;wRf\right)+\left(D_{IBAs}\times \;wIBAs\right)+\left(D_{Pw}\times \;wPw\right) \end{array}$$

Where: wRf = 0.0919, wIBAs = 0.0919, wPw = 0.0748, the values were taken from Steven et al. (11), and *D*_*Rf*_ = the density of rice fields, *D*_*IBAs*_ = the density of important wild waterfowl areas, and *D*_*Pw*_ = the density of permanent wetlands.

**Figure 2:**
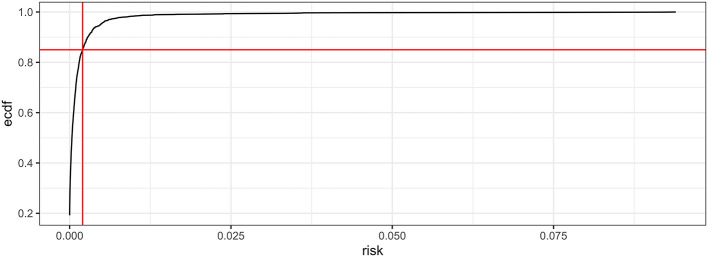
Empirical cumulative distribution function for risk of introduction of avian influenza virus.

The distribution of the data was taken into account to determine the risk categories for monitoring. Figure 2 shows the ECDF, the distribution for the risk of introduction, areas with values ≥0.85 were classified as high risk, all other areas were classified as low risk.

#### 2.2.3 Probability of AIV transmission to domestic animals and poultry

The probability of interspecies transmission depends on the probability of contact between two animal species and the ability of the virus strain to adapt to the new host. This part of the model (Equation 2) is based on the result of the probability of introduction of an AIV by wild birds multiplied by the density of livestock or poultry, stratified by five different production types (Table 1). The analysis was done at the level of the “Consejo Popular”. The following categories were considered:


(2) $$\begin{array}{l} R_{sp,cp}=nRin_{cp}\times \;\left(\sum \;nL_{j1,2,cp}\right) \end{array}$$

➢ Commercial chickens density (number of chickens/km^2^)
➢ Backyard chickens density (number of chickens/km^2^)
➢ Density of commercial ducks (number of ducks/km^2^)
➢ Commercial swine density (number of swine/km^2^)
➢ Backyard swine density (swine/km^2^)

Where: sp, species; j, production type; cp, Consejo Popular; nRin, normalized value of Rin; nL, normalized value of Livestock

A geo-referenced database of commercial farms of all identified owners in the country was available. Based on the centroid of the epidemiological quadrant (1 km^2^) referenced for each farm by the Epidemiological Surveillance Information System (SIVE) of the National Center for Animal Health (CENASA), the location accuracy was at least 0.75 km. The Food and Agriculture Organization of the United Nations (FAO) was used as the source of data for backyard swine density with a resolution of 0.083333 decimal degrees (~10 km at the equator). A full description of the density estimates is given in the companion paper to the ongoing FAO Gridded Livestock of the World project (24). In order to standardize the format of each geographical layer, the raster data were converted into vectors using conversion tools (polygonization and rasterization). The risk categories for monitoring have been taken into account in the ECDF distribution of the data. “Consejo Popular” with ECDF values ≥0.98 were classified as high risk and all other “Consejo Popular” were classified as low risk.

#### 2.2.4 Probability of AIV transmission to farmers or owners

For zoonotic transmission to occur, infected animals must be within the effective transmission range of susceptible humans. The efficiency of transmission will depend on the extent and intensity of contact between animals and humans (commercial or backyard production) and the innate efficacy of the AIV to infect humans (which depends on known or unknown genetic characteristics of the AIV). Both parameters, the contact ratio and the β parameter (Table 1), were obtained based on the methodology of Hill et al. (18), with some adaptations to the specific characteristics of the Cuban livestock system. In the model, the parameters were combined with the probability of transmission of an AIV to domestic animals or poultry to obtain Equation 3. The values used to define risk categories were estimated from the ECDF plot. “Consejo Popular” with ECDF values ≥0.98 were classified as higher risk, all other Consejo Popular were classified as lower risk.


(3) $$\begin{array}{l} R_{sp-fw,j,cp}=nRin_{cp}\times \;nL_{sp,j,cp\;}\times Cr_{sp,j}\;\times \;\beta_{sp,j} \end{array}$$

Where: sp, species; fw, farm worker; cp, consejo popular; j, production type system; nRin, normalized value of Rin and nL, normalized value of Livestock; Cr, contact ratio; β, transmission parameter.

##### 2.2.4.1 Contact ratio of poultry or pigs to farmers or owners

The contact ratio is an indicator of the potential for human exposure to influenza viruses. The relative value assigned to contact between production animals and humans will vary depending on the type of farming system, animal management practices, and worker safety protocols. In commercial production systems, relatively few people will be rearing a large number of animals compared to backyard production, where one or two people will be rearing a small number of animals by hand. There are various assumptions used to establish these ratios, which also vary according to the species and characteristics of the production systems, such as feeding, watering, cleaning, handling, and other tasks involved in animal care and management. Also, regarding the type of housing or confinement systems, the density of animals, and other factors that affect the safety protocols and biosecurity measures on the farm. The values for contact ratios were based on the production manuals for each species in Cuba (25). For the contact intensity model (Cr_j_), the values were normalized, using point values derived from:


$$\begin{array}{l} Cr_{j}\;\sim \;Uniform\left(min_{contact\;rate},\;max_{contact\;rate}\right) \end{array}$$

##### 2.2.4.2 Parameters for the transmission of influenza virus from different livestock production types to farm workers or owners

The epidemiological component of the transmission parameter (β_j_) was generated from published real-world reports (Table 1, Supplementary material) of AI outbreaks resulting in human infections (26–38). The methodology used to obtain β_j_ values was taken from Hill et al. (18). The equation used to estimate β_j_ was:


$$\begin{array}{l} \beta_{j}\sim \;gamma\left(I_{H},\frac{1}{S_{H}\times I_{j}}\right) \end{array}$$

Where I_H_ and S_H_ are the number of infected and susceptible humans and I_j_ is the number of infected animals of production type j in an outbreak.

The information needed to parameterize virus transmission was available in the literature (Table 1, Supplementary material). Humans exposed through routine contact with animals (e.g., farmers and farm workers) were included, but not those involved in post-detection interventions. The number of infected birds is usually not reported in epidemiological reports of such outbreaks, so the total number of animals on infected farms was used as a proxy, except for pig farms, where the total number of susceptible animals or the capacity of the farm was used.

The β values for commercial and backyard poultry were taken from Hill et al.'s (18). For swine (Figure 1, Supplementary material), the formula above was used, informed by values from the literature (26–28). For commercial ducks, there was insufficient real data for birds and humans (susceptible and infected) in the literature to calculate the β parameter, so the average of the β values of commercial and backyard poultry were used.

#### 2.2.5 Probability of human infection

The density of the total human population in each “Consejo Popular” was multiplied by the result of “equation (3)” for each species to estimate the risk of infection for humans. The variable included the total human population divided by the geographical area (inhabitants/km^2^) as recorded by the National Office of Statistics and Information (ONEI) in Cuba. The values used to define risk categories were estimated from the ECDF plot. “Consejo Popular” with values greater than or equal to 0.98 were classified as higher risk, and all others as lower risk. The “Equation 4” was used to obtain the result:


(4) $$\begin{array}{l} R_{cp}=nRin_{cp}\times \left(\sum \left(\;nL_{sp,j,cp\;}\times Cr_{sp,j}\;\times \;\beta_{sp,j}\right)\right)\;\times \;Hp_{cp} \end{array}$$

Where: R_cp_, the risk of transmission to humans in a “Consejo Popular”; sp, species; cp, Consejo Popular; j, production type system; nRin, normalized value of Rin and nL, normalized value of Livestock; Cr, contact ratio; β, transmission parameter; Hp, human population density.

## 3 Results

**Figure 3:**
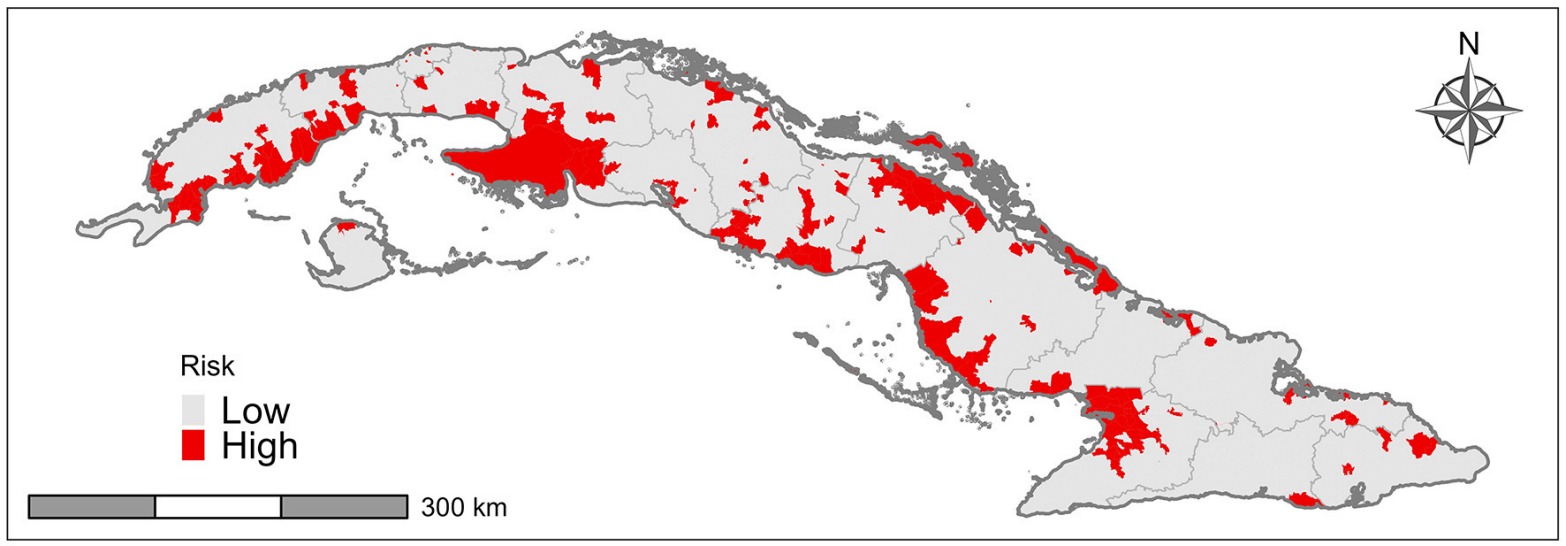
Areas at risk of introduction of avian influenza virus through wild birds.

The model is based on the introduction of AIVs from wild birds into commercial and backyard poultry or swine. The risk of an AIV introduction (Figure 3) was high for 227 (15%) of the 1512 “Consejo Popular” in Cuba. The formation of geographical clusters of high risk “Consejo Popular” was observed in the western region of Cuba due to the interaction of associated risk factors and the contiguity between areas with the same high-risk categories. This region is of great importance for the poultry sector due to its high poultry population density.

**Figure 4:**
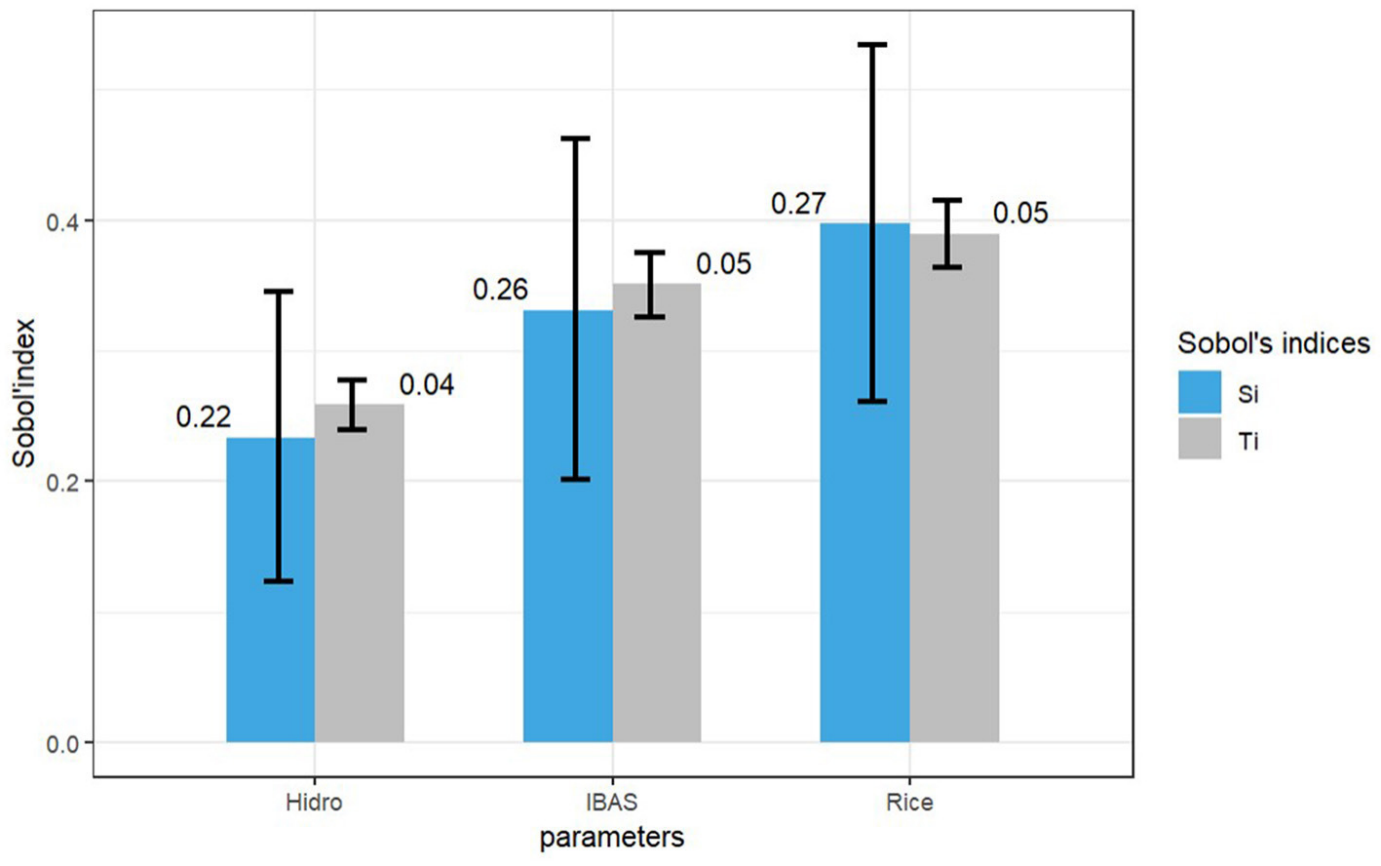
Sensitivity analysis for Equation 1.

The total order (Ti) and first order (Si) sensitivity indices were plotted (Figure 4). Both sensitivity indices had similar values, indicating that there was no significant second order interaction between the parameters modeled to determine the risk of introduction. Values obtained from the difference between the minimum and maximum confidence intervals confirmed that rice fields were the parameter with the highest interaction and sensitivity in the model. This could be due to the geospatial characteristics of the data, for example co-location of rice and IBA areas.

**Figure 5:**
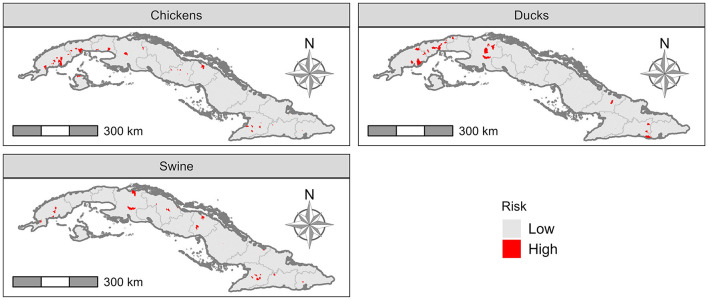
Risk of transmission from wild birds to ducks, swine and poultry.

The high-risk “Consejo Popular” for AIV transmission from wild birds to livestock varied depending on the species (Figure 5). There were 21 (1.4%) higher-risk “Consejo Popular” for commercial ducks, 46 (3.0%) for commercial and backyard poultry and 35 (2.3%) for commercial and backyard swine.

The total (Ti) and first order (Si) sensitivity indices were plotted and presented in the Supplementary material. Both the total and first order sensitivities indicated that there was no significant second order interaction between the parameters. The densities parameter was the most influential parameter (Figure 2, Supplementary material). The density parameters consist of the values of the densities by species (chickens, ducks and pigs) and type of production (commercial and backyard). Density of commercial chickens had the highest value of total order (i.e., contribution to the total variance of the model), followed by backyard swine, which was suggestive of a strong interaction with other parameters.

**Figure 6:**
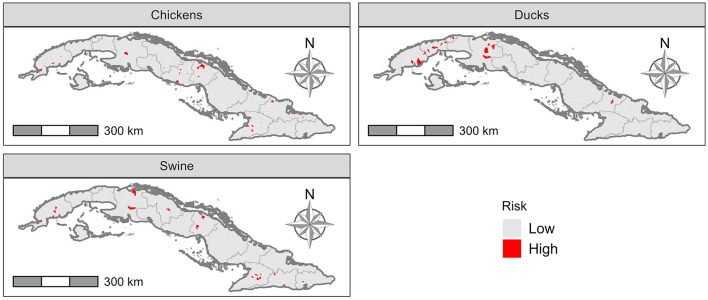
Risk of transmission from domestic livestock to farmer or farm worker.

The risk of transmission from livestock to caretakers is presented in Figure 6. There were 15 (0.99%) “Consejo Popular” with higher risk for commercial ducks, 45 (2.9%) for poultry and 39 (2.6%) for pigs. The results of the sensitivity test (Figure 3, Supplementary material) show that the Rin parameter was the most influential in the output model. In general, all parameters had similar total and first order sensitivity values, indicating that there was no strong interaction between parameters, which could imply that there was no significant second order effect.

**Figure 7:**
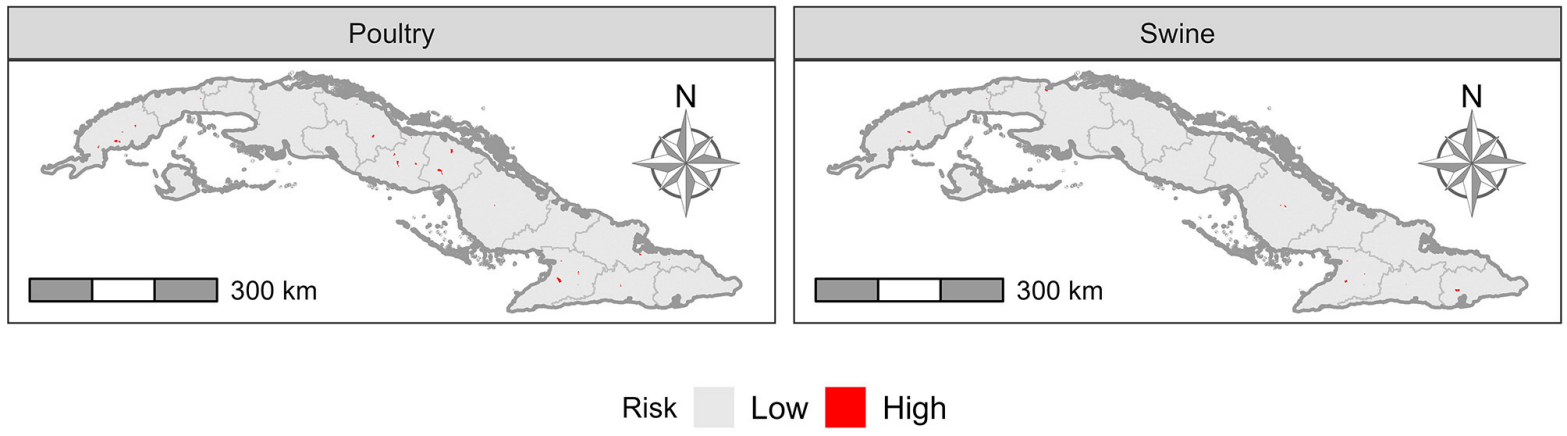
Risk of transmission of avian influenza virus to humans.

Figure 7 presents the risk of transmission of avian influenza viruses from the animal caretakers to the general population. For poultry (commercial and backyard chickens and commercial ducks), there were 39 (2.6%) “Consejo Popular” with a higher risk of transmission, while for swine (commercial and backyard) we identified 24 (1.6%) “Consejo Popular” with increased risk.

**Figure 8:**
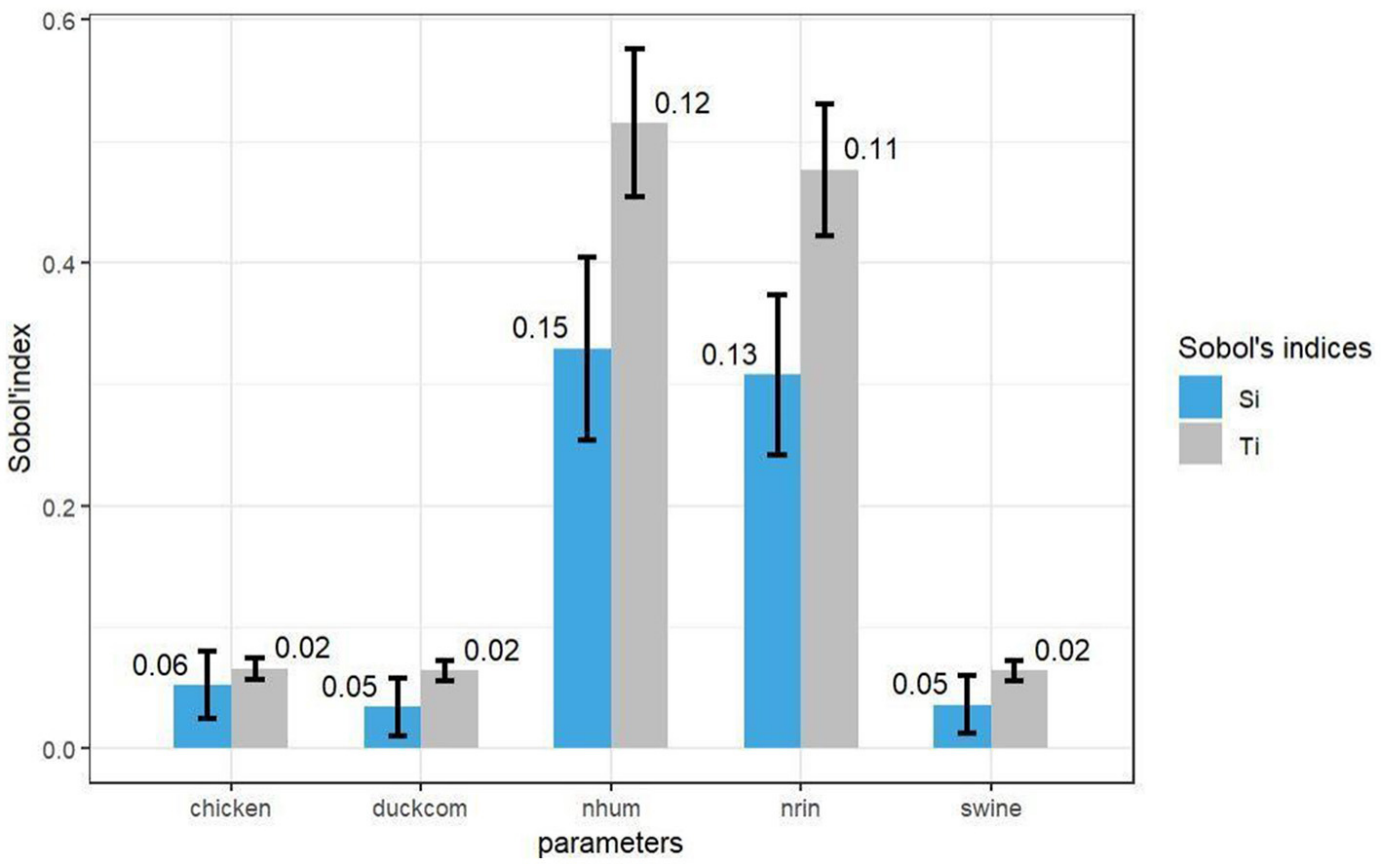
Sensitivity analysis for Equation 4 by species.

Total order (Ti) and first order (Si) sensitivity indices were plotted (Figure 8). The most important parameters for the model output in all simulations performed for the overall model sensitivity analysis were the human population density and the risk of introduction. In the individual sensitivity analysis for each variable included in the model (Figure 4, Supplementary material), the values corresponding to the transmission parameter β for chickens and commercial ducks were the major interaction in the model output. Among animal population densities, poultry and backyard pig populations as well as commercial ducks had the highest interaction. For the contact intensity parameter, the values corresponding to the contact rate between commercial ducks and backyard pigs with breeders were the most influential. Considering the species and type of production separately, the most influential variables for the model output were also commercial ducks and backyard pigs. The latter did not have a high total order value, which means that there is no strong interaction with other parameters. Within the species considered in the model (chickens, ducks and pigs), chickens had a greater first order sensitivity and were considered the most influential species or parameter in the output of the model, together with the aforementioned variables.

## 4 Discussion

This is the first study inspired by a One Health approach to use geospatial modeling and analysis to determine the relative risk of AIV transmission in Cuba. It uses the “Consejo Popular,” which is the smallest administrative division in Cuba, as the unit of resolution. Improving the stratification of geographical areas to identify those at higher risk of AIV transmission will provide health decision-makers with better information to improve risk management through better allocation of resources for prevention and early warning, as well as optimizing the risk-based surveillance system in place in the country. From a One Health perspective, this model allows the identification of “Consejo Popular” where interspecies spillovers may be more likely. These “Consejo Popular” should merit a higher intensity of surveillance to detect early AIV infections before AIV evolution leads to increased pathogenicity or transmissibility within and between species.

Avian influence viruses are expanding their host range, particularly in mammals, often posing a threat to public health (39). Avian influenza A(H5N1) viruses, namely those of clade 2.3.4.4b, continue to diversify genetically and spread geographically. Since 2022, a wider range of wild bird species has been infected worldwide, with adverse ecological consequences and mass die-offs in some species (40). The situation in wild mammals is also of concern, with some species experiencing significant die-offs (41). In 2024, influenza A(H5N1) viruses were detected in neonatal goats at a single facility shared with poultry and in dairy cattle in the United States of America (USA). There have been limited reports of transmission between mammals despite the increase in mammalian infections. Although direct evidence is lacking, large die-offs of marine mammals caused by the influenza A(H5N1) virus occurred; and infections in several fur animal farms in Finland (42) and Spain (43) are consistent with mammal-to-mammal spread in these cases (39). Current evidence from the USA suggests that lateral transmission among cattle has likely occurred. To date, the routes and modes of transmission and the duration of virus shedding in cattle remain under investigation (40). The frequency of transmission from cattle to birds is also unknown. Poultry remain at risk from continued circulation and spillover of influenza A(H5N1) viruses from wild birds.

One of the key advantages of this model is that it could also help to inform risk-based surveillance for individual species. An example of this is the identification of “Consejo Popular” at higher risk of AIV introduction. This information could be of value for the design and implementation of a surveillance component for wild birds, which are considered the main reservoir of AIVs and the main route of entry into Cuba. Surveillance in wild species has not had the desired effect of early detection of outbreaks in other countries, such as the USA and Canada (44). However, when molecular methods are used, it is possible to have a better understanding of which virus strains are circulating and how the disease behaves in wild bird species. This knowledge maybe relevant for risk management.

This model can contribute to improving the current surveillance system by allowing some flexibility and practical adaptability according to current surveillance objectives. The model we described here represents a general framework for introduction and transmission of AIVs in Cuba, but it has the flexibility to incorporate more information. For instance, if information about the circulating AI strains in North and/ or South America are known before these strains were expected to arrive in Cuba. This information could be used to weight certain parameters (e.g., to take into account a predisposition of the virus to infect certain wild birds or certain livestock species) in order to make the model more specific to strains that are expected to arrive in Cuba.

The model estimated the relative risk of transmission of AIVs between humans and commercial and backyard production systems by species, which has value for informing public health surveillance, as well as for improving the active risk-based surveillance system currently in place for commercial poultry. It can also inform the design of a risk-based surveillance system for other species, such as pigs. The identification of areas where risk communication, public education and identification of practices and attitudes of high risk for exposure to AIVs should be a priority (45). Transmission parameters such as Cr and β were constant in the model in terms of spatial distribution, but their value differed according to species and type of husbandry. The intensity of the animal-human contact is used to guide the surveillance of zoonotic diseases in different species (18). Identified areas of high chicken-human contact intensity corresponded remarkably well with HPAI H5N1 cases during the 2003-2004 outbreak in Southeast Asia. This is a clear example of the importance of contact alone (independent of the ability of the virus to infect humans).

Since the beginning of 2021, 28 human cases of influenza A(H5N1) have been reported to the WHO. Of these, 2 have been associated with clade 2.3.4.4b viruses. These cases have been reported to WHO from: China, Chile, Ecuador, the United Kingdom of Great Britain and Northern Ireland, and the United States (40). All human cases, except that the one in Chile, had exposure to infected animals either through participation in outbreak control activities or direct exposure to infected animals in farm, backyard or live bird market environments. The most plausible route of transmission of the Chilean case was through environmental exposure, given the high number of deaths in marine mammals and wild birds found in the area near the patient's residence (46). Among these cases, no human-to-human transmission has been reported. WHO considers the overall public health risk posed by influenza A(H5N1) to be low, and the risk of infection for those exposed to infected birds or animals or contaminated environments is considered low to moderate (40).

Sporadic zoonotic infections have occurred throughout the history of HPAI infections, but with varying risk to humans depending on the combination of genes and mutations present in specific virus variants. While some of the factors that determine higher risk for humans are known, it remains difficult to predict which viruses could trigger more widespread human disease outbreaks, and the ongoing process of viral evolution by genomic reassortment requires regular updates of risk assessment. Actions on protecting animal health and associated consequences for biodiversity is mostly focused on the early detection and control of outbreaks in poultry involving culling and/or regional poultry vaccination. However, this action does not address the fundamentally different pandemic risk to humans in the current situation arising from a panzootic wildlife infectious disease (47).

This model is one of the few analyses conducted in Cuba that is specifically focused on some of the complex interactions of this disease at the human-animal-environment interface, thus providing a unique contribution to our knowledge of the occurrence and transmission of AI in Cuba and worldwide. This model is also one of the few to include pigs in the modeling of influenza virus transmission at the human-animal interface. These species are widely recognized as potential hosts for the generation of novel influenza viruses, and some of these viruses, including pandemic (A) H1N1 2009, have been shown to be readily transmissible between humans and swine (13, 39). However, very few studies have been undertaken to model the spread of influenza at the swine-human-poultry interface.

The model could also be used to design and implement an integrated surveillance system as part of a One Health approach to improve early warning, avoid duplication of efforts and optimize resources for timely diagnosis and rapid response. While high animal and human population densities alone do not imply a high risk of transmission, they could be interpreted as an indicator of various epidemiological processes that are more likely to occur in densely populated areas, such as increased chances of AIV transmission through trade and agricultural activities (48). In the absence of control or prevention measures, the spread of HPAIVs and the occurrence of clinical disease outbreaks will be facilitated in regions where chicken density is particularly high (48). Studies in Indonesia, Thailand, Vietnam and China have linked human infections and poultry outbreaks to several risk factors, including human and animal demographics, i.e., human and poultry population density (49–52), and environmental factors, i.e., percentage of rice paddy area and water sources (53).

The model has some limitations, such as being a density-dependent model without taking into account other factors related to the disease (e.g., prevalence of the virus, virus subtype, species affected, etc.), and some assumptions made for the parameterization of the contact intensity and β parameters. The assumptions made to measure the intensity of contact were based on the values established in the regulations of the Poultry Workers' Payment System, which takes into account the purpose of the farm, the species and the type of production. This is not the case for backyard production, where the values were taken from the literature and expert consultations, which may not be fully representative of the situation in Cuba. The value for the transmission parameter β was also extracted from the literature, and estimated arbitrarily for commercial ducks because of the lack of published data. The lack of information on other factors influencing transmission in geographical area such as transport networks, contact patterns and trade were limitations as well, because many of these factors are considered of high importance for disease transmission even at the global level.

Many of these limitations are due to the lack of necessary data in Cuba, and because HPAIV infections in poultry have not been detected. However, there is evidence that the risk exists, as an AI infection was recently reported in Cuban zoo birds (54) which was successfully contained. Due to the lack of outbreak data, parameterization was done by extrapolating values from the literature or based on expert opinion. Unlike other existing spatial and temporal dynamic models of HPAIVs transmission (55), certain transmission parameters, such as subtype or viral chain characteristics or disease prevalence data, were not considered in the development of this model. These data are scarce even at the global level, which limits modeling and its benefits for the management of the associated risks. However, this limitation in terms of model applicability for epidemiological surveillance was not considered critical.

The increase in the number of emerging, re-emerging and transboundary diseases has highlighted the need to establish effective early warning surveillance systems at both a larger scale and higher resolution (56). This favors real-time processing of disease data with fast dissemination of information to decision-makers, so that rapid prevention and control measures can be implemented against potential outbreaks. The development of a risk assessment model framework can help decision-makers to focus surveillance efforts on regions where pathogen introductions are most likely. It also provides information for the design of risk-based surveillance systems for other species with an integrated and/or unisectorial approach, which can improve early warning, timely diagnosis and allocation of resources for prevention through biosecurity in priority areas.

## Data availability statement

The data analyzed in this study is subject to the following licenses/restrictions: Datasets that are not publicly available can be provided by the first author upon request. Requests to access these datasets should be directed to DM, dnmv.09@gmail.com.

## Author contributions

DM: Conceptualization, Data curation, Formal analysis, Funding acquisition, Investigation, Methodology, Writing – original draft, Writing – review & editing. JB: Conceptualization, Funding acquisition, Investigation, Methodology, Supervision, Writing – review & editing. BD-H: Formal analysis, Methodology, Software, Writing – review & editing. AH: Writing – review & editing, Data curation. MP-A: Writing – review & editing. PA: Conceptualization, Investigation, Methodology, Supervision, Writing – review & editing. LC: Conceptualization, Funding acquisition, Investigation, Methodology, Supervision, Writing – review & editing.
