# Supplementary figures and images for: Modeling transmission of avian influenza viruses at the human-animal-environment interface in Cuba
Source: Front Vet Sci. 2024 Jul 11;11:1415559. doi: 10.3389/fvets.2024.1415559 (PMC11269842; doi:10.3389/fvets.2024.1415559)

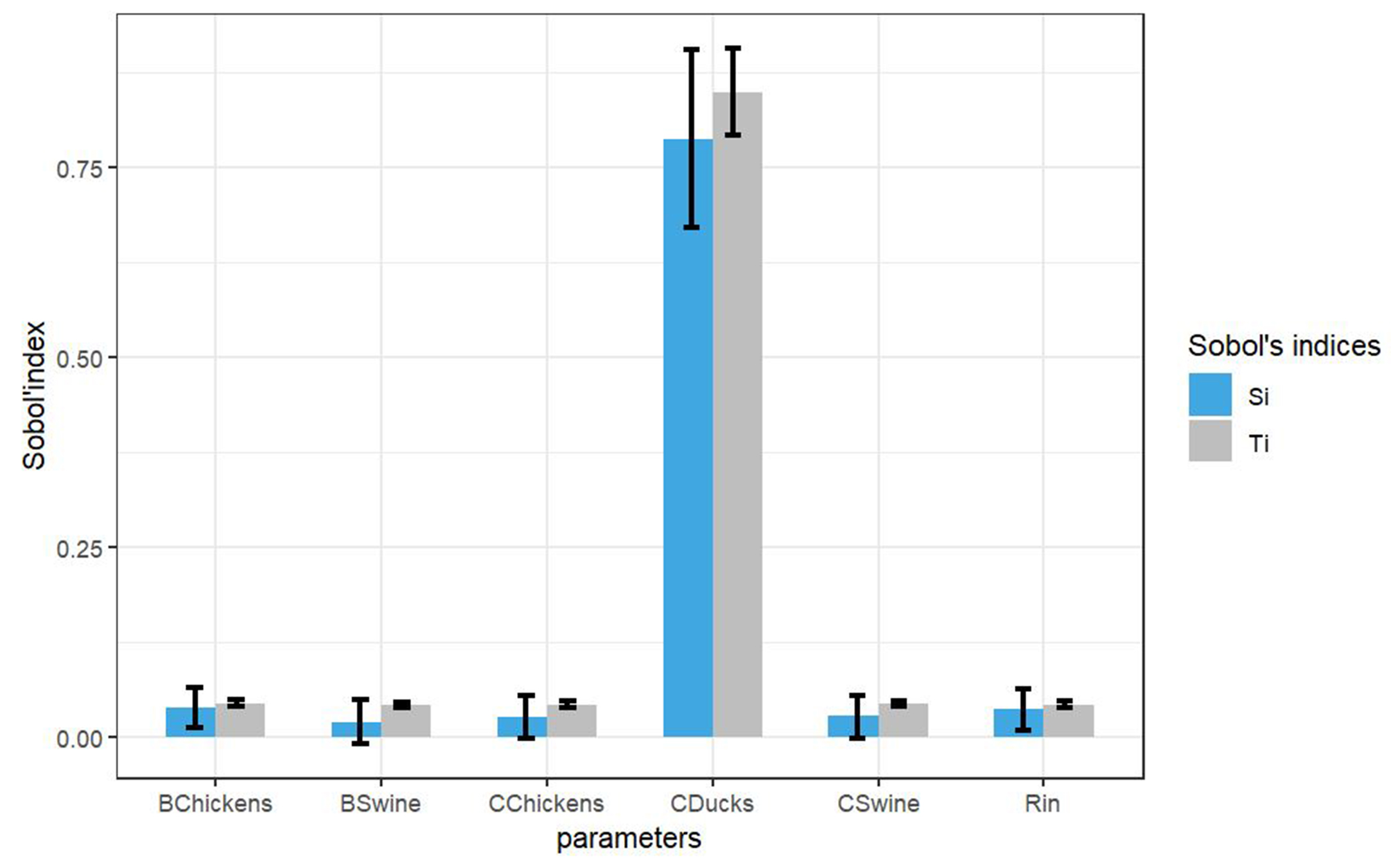

Supplement: Supplementary file 1 [file Image_1.JPEG]

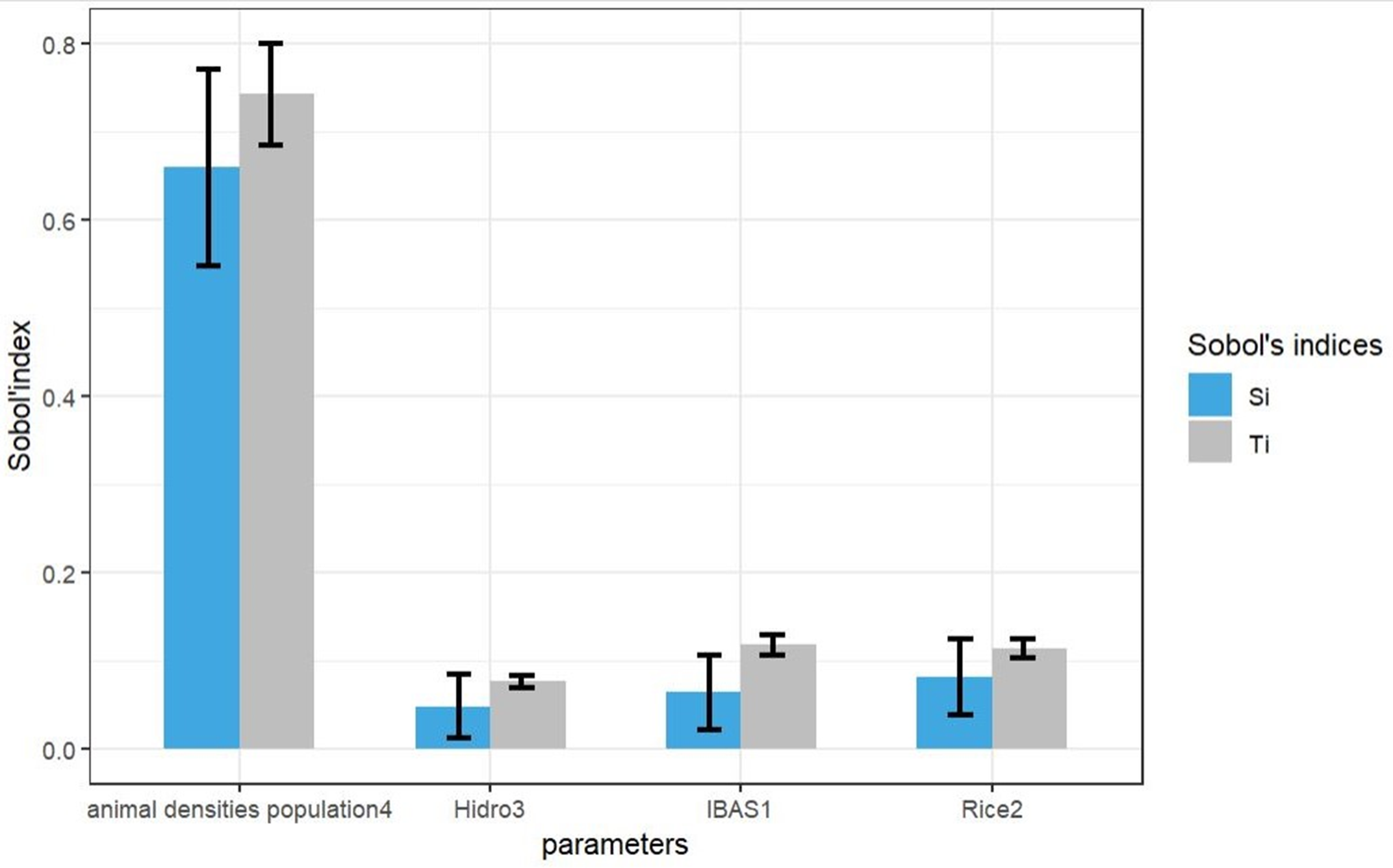

Supplement: Supplementary file 2 [file Image_2.TIFF]

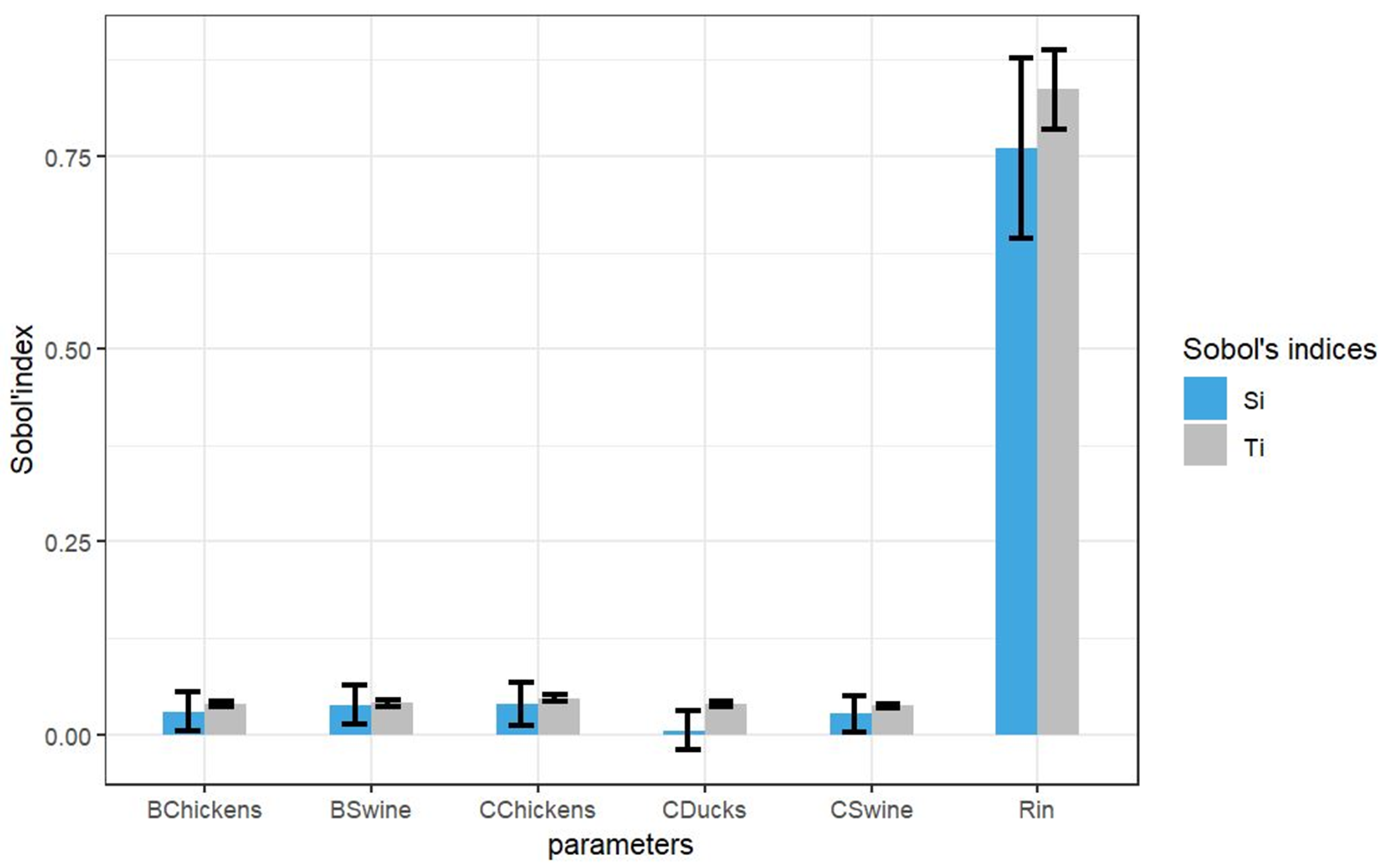

Supplement: Supplementary file 3 [file Image_3.TIFF]

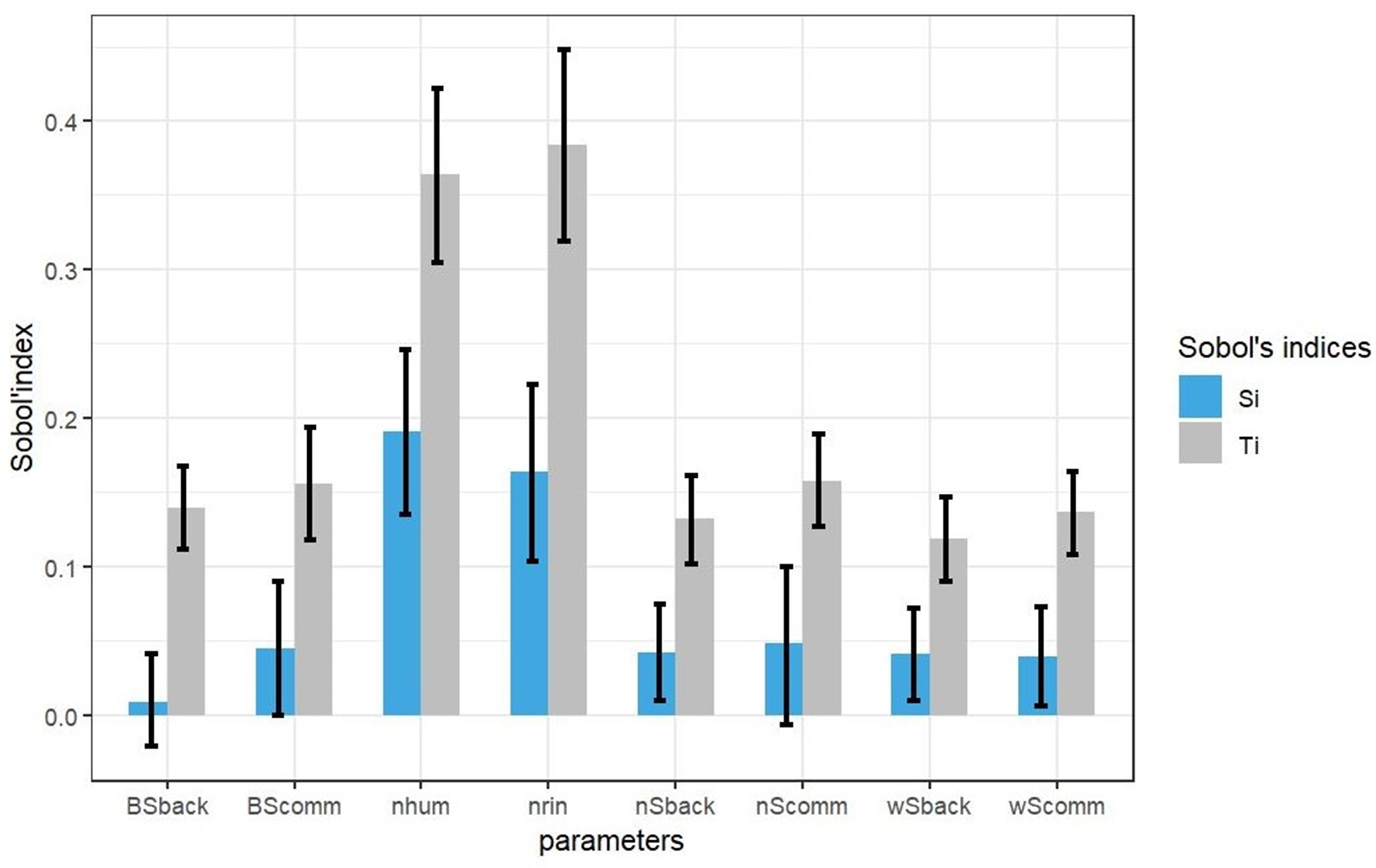

Supplement: Supplementary file 4 [file Image_4.TIFF]

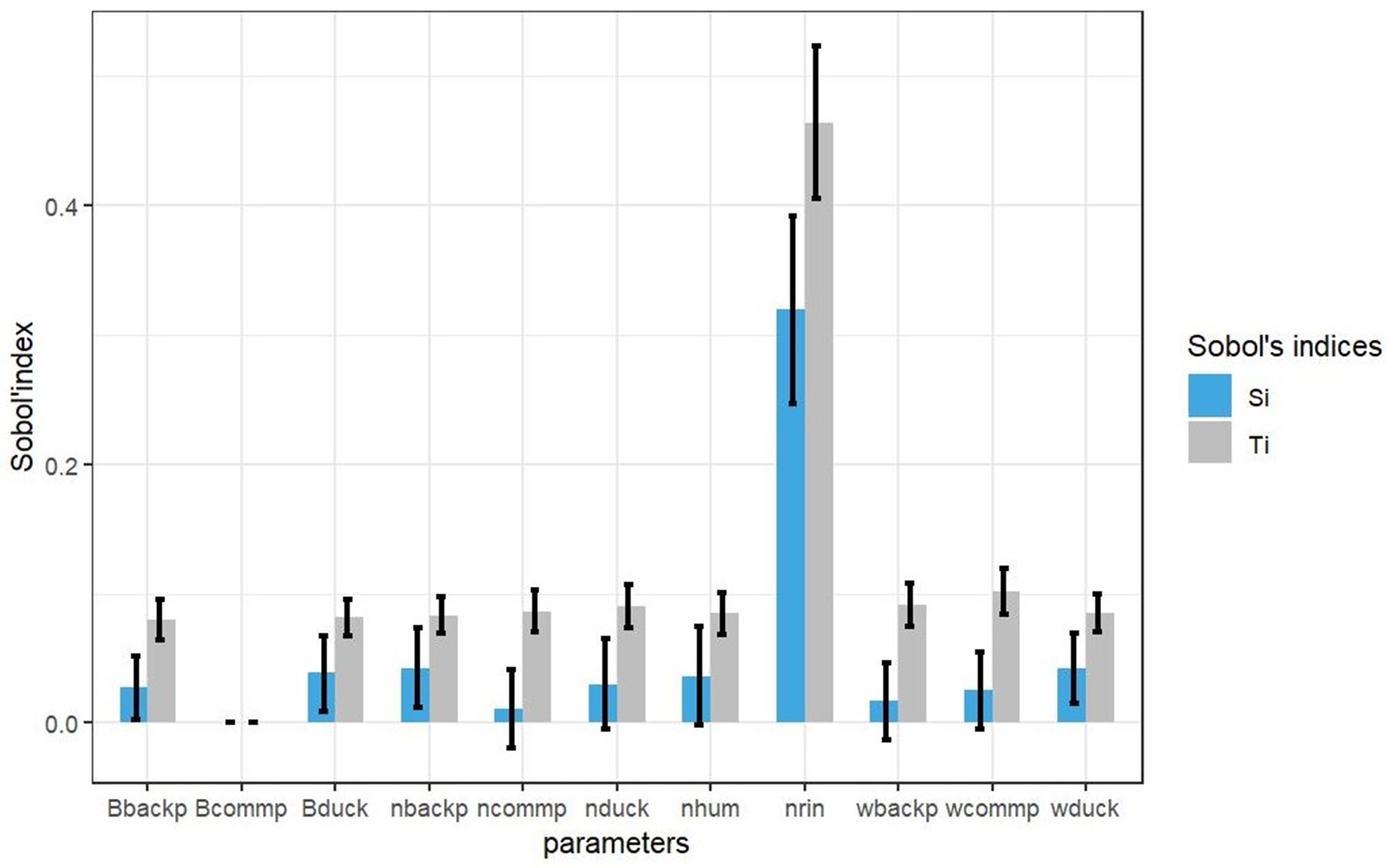

Supplement: Supplementary file 5 [file Image_5.TIFF]
